# War Typhus and Dysentery

**Published:** 1871-07

**Authors:** Rudolf Virchow


### Selections.

### WAR TYPHUS AND DYSENTERY.

By RUDOLF VIRCHOW.

(Kriegstyphus und Ruhr. Von R. Virchow. *Virchow's Archiv.* Bd. LII. S. 1. January 16, 1871.)

The January number of *Virchow's Archiv.* contains an essay by the editor, on the fever and dysentery of the German army in France last fall and winter. Aside from the fact that everything relating to this wonderful campaign is just now of interest, this paper contains so much matter for consideration that we have thought a rather full abstract of the contents would be acceptable.

The author begins by relating that at the commencement of the war a series of great and apparently decisive battles rapidly carried the victorious army into the interior of the enemy's country. Numerous dead and wounded were left behind, but the general health of the invaders remained good, until siege operations succeeded the battles in the open fields, and great numbers of men were shut up in Metz and Paris, by vast investing armies. Then pestilence broke out. First around Metz, where dysentery soon appeared, followed by typhus. The improvised hospitals in schools and halls, as well as the newly-built barracks, became encumbered with the sick, and endless railroad trains carried them to the rear. By the middle of October fifty thousand had been sent. The besieged, too, suffered greatly. When the fortress surrendered, twenty-five thousand sick and wounded were found.

The news from Paris at the time of writing was less detailed, but dysentery and typhus had already appeared.

These two chief war diseases, therefore, demand attention at the present time; and first, of typhus. Spotted typhus (the typhus of English and American writers) must be distinguished from abdominal typhus (the typhoid fever of English and American writers). The Napoleonic wars were followed by terrible epidemics of spotted typhus, but two generations sufficed to throw the old knowledge into the background; and although in France, and especially in England and Ireland, repeated sad experiences maintained a knowledge of the distinction between the two forms, in Germany it became the general practice to call abdominal typhus shortly typhus, and to regard belief in any other form as a mere superstition. The hunger typhus in Upper Silesia first checked this error, the characteristic lesions of abdominal typhus being absent, and the Crimean war produced a general reaction of opinion. It was then clearly shown that petechial or spotted typhus was not merely the hunger epidemic, but the true prototype of field and camp typhus. Hence, when news of typhus first came from Metz, it was natural to fear the disease might be of this formidably contagious character.

But although on former occasions war typhus has generally been spotted typhus, yet this has been replaced by the abdominal form in certain epidemics, as, for example, in that of Mayence, 1813-14. In a little pamphlet, on his recent journey to Metz with a sanitary train of the Berlin Hülfsverein, Virchow stated abdominal typhus to be the prevailing form there. Later observations, especially since the surrender of Metz, have confirmed this opinion. At Giessen, it is true, a few cases, supposed to be spotted typhus, were reported among the female nurses; but abdominal typhus shows so many varieties, that we must be careful in judging with regard to single cases; and "especially the admixture of the malarial element, which has shown itself actively during this war by numerous cases of intermittent fever, confuses the picture of the so-called normal course of typhus in a manner which is sometimes quite deceptive."

Considerable confusion of ideas exists in certain quarters with regard to the eruption of spotted typhus. The name petechial typhus has led some to regard "true petechiæ" as the characteristic eruption. This word petechia, as used at present, has no longer the same meaning as when first introduced into scientific terminology by Fracastoro. The comparison with flea-bites, which gave origin to the name, readily led to confusion of ideas, for a recent flea-bite, in the midst of the little red spot due to capillary hyperæmia, presents a small, dark, central area of hemorrhage around the point of puncture. The first disappears after a few hours, the latter remains longer; so that a recent flea-bite resembles the spots of roseola, and this was the sense in which the name petechia was originally employed; an older flea-bite resembles the hemorrhagic spots called petechiæ in modern times. These resemblances are so great that they may even complicate diagnosis; though the fact that roseola spots have not the central dot of hemorrhage seen in recent flea-bites, and that older ones are smaller and have more abrupt edges than is usual with petechiæ, will generally enable them to be distinguished.

During the seventeenth and eighteenth centuries, the notion that hemorrhagic spots were characteristic of typhus gained ground. They were supposed to be the expression of a putrid condition of the blood, and the name putrid fever was employed to designate all the forms of typhus. But, in fact, these hemorrhagic spots occur not only in both spotted and abdominal typhus, but also in numerous other diseases, and there is nothing either in their character or the time of their appearance which is significant for diagnosis.

The eruption of merely hyperæmic spots has much more diagnostic value. These may be scattered and isolated, in the form of roseola, or more numerous, larger and closer together, resembling measles, with which they may even be confounded. Virchow described this eruption in connection with the épidemic in Upper Silesia (*Virchow's Archiv.*, vol. ii., p. 187; vol. iii., p. 165), but protests, that with all his opportunities for the observation of fevers, he is unable to admit any such constant differences between the roseolar eruption of abdominal typhus and that of spotted typhus, as are laid down by many writers.

"In both cases we have essentially a roseolar exanthem, consisting of multiple spots of capillary hyperæmia in the skin, and not limited to the hair bulbs. At first the redness disappears entirely on pressure, but at a later period the color becomes darker, probably through a partial escape of the hæmatin of the blood-globules, and its imbibition by the tissues; it then no longer disappears under the pressure of the finger. In spotted typhus the exanthem is well marked and wide-spread. In connection with the diagnosis, I would expressly remark that in our recent epidemics I have often seen it on the face, even on the forehead, as well as on the palms of the hands and the soles of the feet, so that a limitation of the eruption to the trunk and the adjoining portions of the extremities cannot be regarded as a constant diagnostic mark from measles."

"In abdominal typhus the exanthem is usually less marked, the spots are scattered, and limited to certain regions, especially the upper portion of the abdomen and the lower part of the chest. But there are also unmistakable cases of abdominal typhus in which the exanthem is much more decided; the single spots are then larger, more strongly colored, and spread more widely over the body. If we consider, finally, that it not unfrequently happens that in spotted typhus the exanthem is slight, and the spots scattered, it will be understood, in the first place, that both abdominal and spotted typhus are alike entitled to be considered as exanthematous diseases; and, in the second place, it will be seen that the exanthem alone, without taking the other symptoms into consideration, is not decisive, and cannot be strictly regarded as a pathognomonic symptom."

Virchow next mentions that some cases of recurrent fever have been reported among the German troops in France; states that though he has seen petechiæ, he has never seen a roseolar or measle-like eruption in this disease, and discusses at some length the question of the occurrence of recurrent fever during the hunger epidemic of 1847–8 in Upper Silesia.

He then proceeds to suggest, that as the pathological changes in the intestine and the mesenteric glands, which are characteristic of abdominal typhus, can only be distinguished with certainty after death, autopsies are always of great importance in deciding the character of any epidemic. This has been done during the present war in the cases of a number of soldiers who died after their return to Berlin. "Extensive medullary infiltration of the lymphatic apparatus of the ileum, and to some extent of the colon, ulcers, and cicatrices, were found. We have during life no indisputable signs of these alterations. The diarrhœa, and the development of gas in the intestines, are certainly noteworthy, especially when fluid and gas occur at the point chiefly affected, the lower ileum and the cœcum, developing the well-known ileo-cæcal gurgling. But this gurgling is not specific, and may occur in the same way in spotted typhus."

It is his opinion that the notion that the diarrhœa of abdominal typhus is chiefly dependent upon the formation of ulcers is erroneous. Both in this disease and in tubercular ulceration of the intestine, the diarrhœa is dependent upon the co-existent intestinal catarrh, traces of which are found in the autopsies far beyond the portions of the intestines which are ulcerated, and no connection can be shown to exist between the severity of the diarrhœa and the extent of the ulceration. In abdominal typhus, then, we must consider two independent affections. "The catarrhal affection of the mucous membrane, which often involves the whole small intestine as far as the stomach, and the progressive alteration of the lymphatic apparatus, which involves the solitary follicles and the patches of Peyer, as well as the mesenteric glands."

In the spotted typhus of Upper Silesia, Virchow observed in some cases severe intestinal catarrh, attended by simple swelling of the follicles. He has also observed diarrhœas of great severity in the recent epidemics of spotted typhus in Berlin. In some of these cases, the discharges were found to contain large quantities of well-preserved intestinal epithelium adhering in shreds, just as in cholera. In the cholera epidemic of 1866, the presence of epithelial cells in the discharges was questioned. Virchow, however, has seen them in great abundance in cholera (*Medicinische Reform, 1848, p. 28*), and explains their absence in many cases by supposing them to be decomposed in the large intestine, a circumstance which would be still more apt to occur in either abdominal or spotted typhus.

But not merely is it important to separate the question of the diarrhœa from that of ulceration; the ulceration itself is not to be looked upon as an invariable concomitant of abdominal typhus. Virchow mentioned, as early as 1848 (*Virchow's Archiv.*, vol. ii., p. 244), that "a resorption of the exudate in the stage of medullary infiltration is possible." He is now disposed to believe that resolution is the normal termination of the affection of the intestinal follicles in abdominal typhus, and that it is an error to suppose that ulceration is of regular or necessary occurrence.

Not as he thought in 1848, that we have to do with a simple exudation; he has since shown that the medullary infiltration of Peyer's patches, and the solitary follicles, consists from the first in an increasing number of lymph cells quite like the normal gland cells of the lymphatic apparatus. This accumulation of elements takes place at first in the follicles, afterwards in the tissues which surround them. The alteration is therefore quite similar to that which occurs in the mesenteric glands, where it has long been known that resolution takes place. The precise mechanism of the process of resolution is not fully made out. "Certainly, fatty metamorphosis may be observed, but it seldom obtains a considerable height, and it is therefore questionable, whether besides this change, or even without its occurrence, the swelling is not simply relieved by one cell after another being carried off in the lymph stream." But whatever the details, the process occurs in the glands of Peyer and the solitary follicles, as well as in the mesenteric glands.

If resolution does not take place, the medullary infiltration passes into the cheesy metamorphosis. The product is the well-known typhus slough, not an ulcer; the ulcer only originates on the separation of the slough. The typhus slough is originally dry, firm, and of a yellowish-white color; but it rapidly alters, partly by decomposition, partly by the action of the intestinal contents. Bile pigment may tinge it yellowish or brownish; iron given as medicine may stain it black.

In the mesenteric glands, the formation of the typhus slough is rarer. When it occurs, the cheesy mass remains longer unaltered than in the intestinal follicles. The condition, in fact, resembles what may be seen in scrofulous or tubercular lymphatic glands. The whole gland does not usually become cheesy, and even in the more severe cases the process is usually limited to a few glands near the junction of the ileum with the cœcum. After a time, suppuration occurs around the slough, and a furuncle-like gland-abscess results, which may open into an adjacent knuckle of intestine, or may become encapsuled.

In the case of the intestinal follicles, the slough may be as extensive as the original medullary swelling, whether in the solitary glands or the patches of Peyer; but in the latter case the ulcers are usually smaller than the patch, so that there may only be one or several small ulcers in a single patch. In a general way it may be said, that the number and size of the patches in which the medullary swelling occurs, bear no proportion to the number and size of the ulcers. The ulcers are most frequent in the patches of the lower part of the ilium; but even these sometimes escape, and higher up the intestine this is still more frequent. If this is true even in fatal cases, may we not infer that in the non-fatal cases, especially in those in which speedy recovery takes place, resolution of all the patches frequently happens? Is it wise to assume that ulceration followed by cicatrization is the usual mode of cure? Autopsies of the so-called walking cases show that it would not always be safe to assume that no ulceration has taken place because the case appears to be mild; but is it not probable that the resolution which we know occurs in some patches in almost every case, more frequently occurs in all than has been hitherto believed?

At the seat of war there has been repeated mention of transitions between abdominal typhus and dysentery. Such expressions originate in a misunderstanding. We must not confound the names of diseases based on a clinical experience with those which are founded on pathological anatomy. Dysentery, like apoplexy and pertussis, is a clinical designation. It implies discharges from the bowels accompanied by tenesmus. It has been referred to inflammation of colon and rectum, but sometimes a large part of the ileum is involved also, and it is not every colitis or proctitis that is dysenteric.

Schönlein grouped dysentery with angina gangrenosa, croup, hospital gangrene, and septic metritis. Rokitansky went so far as to speak of a puerperal uterine dysentery. Here we see anatomical notions confusing the clinical. Rokitansky only meant that in these cases the lesion of the mucous membrane of the uterus was similar to that observed in the dysenteric intestine.

Virchow taught a long time ago that the intestinal lesion in dysentery was in many cases similar to that which Bretonneau called diphtheritis when the mucous membrane of the throat was affected, and that a similar process occurred also in cholera, as well as in the enteritis which sets in above intestinal strictures. Since then, many speak as if every dysentery were due to diphtheritic inflammation—as if diphtheritis of the colon or rectum was the correct anatomical expression for what the clinicists call dysentery. He protests that he feels free from the blame of having originated this error. In fact, he began his account of dysentery by stating (*Virchow's Archiv.*, vol. v., p. 348) that a catarrhal dysentery may, under certain influences, be aggravated into a diptheritic dysentery. For simple catarrhal inflammation of the mucous membrane, often of great extent, occurs in dysentery as well as in typhus. In typhus it affects chiefly the small, in dysentery the large intestine.

The discharges produced by the dysenteric catarrh contain commonly, besides mere fluid matter, those thick glairy masses called mucous when they are transparent, croupous or fibrinous when they are opaque. This is the so-called "white dysentery;" in the "red dysentery" the discharges only differ by containing more or less blood. Both kinds of stools may occur without any ulceration or erosion of the mucous membrane, and the hemorrhage may be simply due to the rupture of congested capillary vessels during the spasmodic contractions of tenesmus. Hence, the amount of blood discharged is often closely related to the degree of tenesmus.

The subacute and chronic forms of catarrhal dysentery readily become complicated with follicular ulceration of the colon. Bamberger is right in distinguishing this form of ulcerative colitis from diphtheritic inflammation. But he shares an old error of the school of Prague when he speaks of the swollen follicles as exhibiting at times clear transparent contents, for such cysts, which belong only to the later stages of chronic dysentery, originate in the coalescence of the follicles of Liebekühn. They are described in Virchow's book on tumors (vol. i., p. 243.)

The primary lesion in the follicular ulceration of dysentery is the conversion of the solitary follicles into little abscesses; these on their rupture leave small cavities, which manifest a tendency to burrow, and thus to coalesce, forming often large ulcers, and sometimes causing the separation of portions of mucous membrane of considerable dimensions.

Bamberger adheres to another error of the school of Prague. He regards those little lumps in the stools, which resemble cooked sago or frogs'-eggs, as the secretion of the diseased solitary follicles. Virchow, however, has shown (*Virchow's Archiv.*, vol. v., p. 329) that the iodine reaction generally demonstrates these lumps to consist of undigested, starchy matters. Where they are truly mucous masses, however, they must proceed from the glands of Lieberkühn, not from the solitary follicles.

It must be added that follicular ulceration is not peculiar to dysentery; it occurs also in other forms of intestinal catarrh, as, for example, in that of phthisis.

We have next to consider the diphtheritic process already mentioned as occurring in dysentery. The pseudo-membranous patches are greyish-white, or grey if they occur on portions of the mucous membrane affected simply by catarrhal inflammation. If they occur on portions of mucous membrane into which hemorrhagic effusions have taken place they are brownish-red, blackish, or greenish-black. In either case they may be stained variously by the intestinal contents.

The diphtheritic layer may be superficial, or may extend deeply into the mucous membrane. Its depth will determine the depth of the subsequent ulcer. Ulceration is the constant result, the diphtheritic layer being separated as a slough by a suppurative process. These ulcers are unlike the follicular ulcers of catarrhal dysentery, and they do not burrow. Yet it is to be noted that the diphtheritic process sometimes involves the follicles, producing follicular ulcers, along with the diphtheritic destruction of tissue.

From the foregoing sketch it will be understood that we cannot substitute the anatomical designations of either of the above lesions for the clinical term dysentery, which is based on symptoms and etiology, and embraces both the catarrhal and the diphtheritic form. The former is the less severe variety, and it is probable that the diphtheritic process only sets in in portions of the mucous membrane which are already affected by catarrh. Moreover, simple catarrhal inflammation of some portion of the intestine always co-exists with diphtheritic alteration of others. This is shown by the fact that the discharges from the bowels continue—for the diphtheritic patches secrete nothing, and only discharge a little pus after the sloughs begin to separate.

It will now be understood that there is no reason why abdominal typhus and dysentery may not co-exist in the same person; the diphtheritic form of dysentery is most apt to occur in such cases, just as diphtheritic sore throat sometimes sets in during abdominal typhus. In fact, all three processes may co-exist. Usually the dysenteric process sets in during the latter stages of abdominal typhus. Such cases are, however, comparatively rare, and Virchow declares he does not know of a single instance in which abdominal typhus has developed itself in a patient already suffering with dysentery.

He next proceeds to compare what he has learned of the fever and dysentery of the German armies in France with what occurred during the late war in America.

"It is worth while to state, in the interests of the comparative pathology of war epidemics, that the conditions observed in the present war approximate in many particulars those of the late American war. There also spotted typhus was almost never observed, and the reporter of the medical staff, Woodward, was even inclined to refer the few cases reported under this designation to inaccurate diagnosis. (*Outlines of the Chief Camp Diseases of the United States Armies, Philadelphia, 1863*, p. 153.) In his later report, which forms a part of the contents of the renowned Circular No. 6 of the Surgeon-General's Office (p. 113), he admits a limited number of cases of true typhus, especially among prisoners of war. This number, however, was so small that Bartholow, who contributed the report on camp fevers in the medical section of the sanitary memoirs of the war of the rebellion, collected by the United States Sanitary Commission, expresses his opinion that it is questionable whether mere overcrowding and insufficient ventilation are sufficient in themselves to generate typhus. He insists particularly upon the absence of typhus in the notorious Andersonville prison. For the rest, cases of 'spotted fever' occurred, it is true, in many regions in America, but the report of Hunt (*Contributions*, p. 390) states that those were really cases of cerebro-spinal meningitis." While spotted typhus was rare, abdominal typhus and dysentery were wide-spread in the American army. In circular No. 6, the number of cases of abdominal typhus for the first year of the "war is reported as 21,977, with 5,608 deaths; the total being for the second year, 31,374 cases and

10,467 deaths; the total being 53,351 cases and 16,075 deaths. During the first year, 215,214 cases of acute and chronic diarrhœa and dysentery were reported, with 1,194 deaths. Since the limits between diarrhœa and dysentery cannot be very sharply drawn in medical reports made often from different points of view, and since it appears from the statements of American physicians, and particularly from the pathologico-anatomical reports of Woodward, that a large part of the cases of so-called diarrhœa were really dysenteries, we may assume that in spite of the enormous number of diarrhœa cases reported, dysentery was nevertheless an exceedingly prevalent affection. During the second year of the war the number of cases of diarrhœa and dysentery amounted to 510,461, with 10,366 deaths. The total for the two years was therefore 725,675 cases and 11,560 deaths. More than one-fourth of all the cases of sickness belonged to this category. On the other hand, the mortality from abdominal typhus was absolutely greater, although the number of cases was about fourteen times less."

"The secession war also showed a similarity to that of Germany with France, in the gradual manner in which abdominal typhus increased in frequency and severity. It is important to the future solution of etiological questions to draw the attention of physicians now to this agreement."

Next, with regard to the etiology of the diseases under consideration. Hippocrates has mentioned the prevalence of dysenteric diarrhœa and protracted quartan fevers during the summer. Observers of every age, especially in hot countries, have laid stress on the connection of dysentery and diarrhœa with malaria.

This has perhaps nowhere occurred to such an extent as in North America, where the medical historian of the war has even gone further, and established a special group of fevers, first described by Woodward (*Outlines*, p. 74) under the name of typho-malarial fever. But it seems to me that we should be very cautious in this direction. Certainly malaria, as I formerly mentioned, exercises a predisposing influence on the development of dysentery, since nearly all febrile conditions occurring in the warm or mild seasons, in swampy regions, or during the prevalence of intermittents, are complicated with "catarrhal affections of the mucous membrane of the intestines. But the determining must be distinguished from the predisposing influence. That mere exhalations from the soil—and as such malaria must be considered—are capable of generating dysentery or typhoid disease, is exceedingly questionable." (*Virchow's Archiv.*, vol. v., S. 354.)

More importance is attached by Virchow to the influence of impure drinking water in producing these disorders. His opinions as to its relations to typhus are given in a recent paper (*Virchow's Archiv.*, vol. xlv., p. 294). With regard to dysentery, too, it is important. In 1770, a hundred years ago, an epidemic of dysentery occurred in a regiment stationed at Metz, which was clearly traced to the use of water from wells contaminated by adjacent latrines (*Guilhaumon. La guerre et les epidemies*, p. 64). The Romans supplied Metz with pure water from a distance, by means of a great aqueduct, the ruins of which are still seen. A new aqueduct performing the same functions was interrupted during the recent siege by the German troops. The notion that the soil and water around Metz are favorable to the development of fever and dysentery is not without support from history.

Just before Metz was taken from Germany, in 1552, the Emperor Charles V. besieged the place with 80,000 men. During the months of November and December, a third of his army melted away, chiefly from dysentery, scurvy, hospital gangrene, and camp fever. In the city, things grew so bad that it was commonly supposed that the surgeons had poisoned the bandages, till Ambrose Paré, who visited both the imperial camp and the city, dissipated the suspicion. After the siege was raised, a terrible epidemic of typhus broke out in Metz, and devastated the already desolated valley of the Moselle, from Pont-à-Mousson to Thionville (*Guilhaumon*, loc. cit., p. 36).

Again, after the battle of Valmy, September 20, 1792, when the allied German armies retreated before the French, a very contagious epidemic of dysentery broke out among them, and involved also the French army and population. It was known as the "Courrée Prussienne." The retreat commenced in Champagne, and lasted twenty-two days. The first cases, however, filled the hospitals of Verdun and Longwy, not those of Metz. Typhus followed, but appeared first in the Department of the Mass, and the districts of the Moselle, the Meurthe, and the Ardennes; everywhere the hospitals were crowded. In the sequel, typhus prevailed for two years in Metz, 64,413 men being received into hospitals there; 4870 died.

But these accounts hardly furnish the details for an exact investigation into the causes of the pestilence. They show, however, that at least no one place can be held responsible for their origin, and this is doubtless one reason why some investigators give prominence rather to the supposed influence of season.

"Nearly all the epidemics of typhus in or near Metz commenced late in the summer or in the fall. Woodward (*Circular No. 6*, p. 112) mentions the 'autumnal' character of the North American camp fever, as a corroboration of his view of the prevalence of a malarial element among the causes; and diarrhœa and dysentery were so much more frequent in the Union armies during the summer and autumn, that the definite influence of the time of the year seemed indisputable. (*Circular No. 6*, p. 120.) In the present campaign the appearance of dysentery was not confined to any special malarial region. The first news of it came from Saar'rück. The hospitals of Saarlouis and along the line of railroad from Remilly soon filled, and the army was already suffering from dysentery when it came before Metz. After the disease made its appearance, contagion may have played a considerable part; but its first appearance must be referred to other circumstances. Nothing, then, is left, but to admit the effect of injurious alimentary and thermal conditions."

Virchow then proceeds to sketch the irregular and often improper food and drink of the troops, their exposure to cold nights after hot days, to inclement weather without shelter, repose on the damp ground, and the like. But he suggests, that besides all such causes, some local influence, acting on particular parts of the intestines appears probable, and thinks this may perhaps be found in constipation with fœcal accumulations. He refers to the local diphtheritic processes, and the deep and numerous follicular ulcers often seen just above strictures of the intestines. Perhaps the ammonia evolved by the decomposition of the fœcal matters is the immediate irritant. (See *Virchow's Archiv.*, vol. v., p. 356.) In Bright's disease, where considerable quantities of urea occur in the intestinal secretions, and rapidly decompose, extensive intestinal diphtheritis is common. Ammoniacal decomposition of the urine is known to produce catarrh of the bladder, ureters, and pelves of the kidneys. In vesico-vaginal fistula, diphtheritis of the vagina is common.

He does not insist upon this view to the exclusion of all co-operating influences; malaria, bad water, the cachectic condition which the Americans name "the scorbutic taint"—doubtless all exert their influence—but he thinks it improbable that true dysentery ever arises, unless, besides these, either contagion or fœcal retention exists as the immediate cause.

In abdominal typhus, contagion is probably of less significance, but its possibility cannot be excluded. The contamination of the soil by decomposing animal matters, and the emanation thence of a peculiar poison acting either through the air or the drinking water, is generally regarded as the cause. Overcrowding of habitations, deficient nourishment, season, place, etc., may co-operate, but are probably of secondary importance, though in a general way, whatever can evoke an intestinal catarrh may act as an accessory cause.

"The casual momenta are nowhere more readily brought together than in a besieging army, especially where the siege is of long duration. The scanty and irregular nourishment, the want of shelter, the exhausting service—above all, the uncleanliness which necessarily increases from week to week, affect the besiegers much more severely than they do the besieged." It is not surprising, then, that this was the case at Metz in 1552 as well as in 1870. But in protracted sieges the condition of the besieged becomes the worse in the sequel, especially if the place is overcrowded, or occupies an unhealthy site. Under such circumstances devastating pestilences may break out, as happened at Dantzic, at Torgau, and at Saragossa.

So little is known positively about the causes of abdominal typhus, that our prophylaxis is uncertain. Among the *personnel* of three of the sanitary trains from Berlin to Metz, of which only Virchow knew the particulars, cases of abdominal typhus of a severe form occurred. The circumstances are recounted in detail, and the conclusion drawn that they warrant the inference of contagion.

The influence of contagion in the production of spotted typhus and of recurrent fever is clearer. Every time these diseases have become epidemic in Berlin, of late years, they appear to have been imported from the provinces, and the circumstances have not been such as to show clearly the influence of either famine or overcrowding, while they have rendered contagion highly probable. It is true that typhus has often occurred in prisons and fortresses; but the case of Andersonville shows that we must not reason too hastily with regard to such instances. Moreover, in the majority of those cases in which war typhus has occurred in camps and fortresses on the continent of Europe, it can be traced directly to contagion, and overcrowding has served rather to favor the spread of the poison than to originate it.

It can no longer be disputed, at the present time, that the Sclavonic countries are the ever-overflowing source of spotted typhus, as they are also of the rinderpest. This is the secret of the supposed intimate connection between the two diseases. The present war has disabused our minds with regard to such a supposition. The rinderpest was carried everywhere by the cattle brought for the army from Podolia and Galicia; it spread through Pomerania and Mecklenburg, through Mark and Saxony, until it reached France. Yet there has been no appreciable number of cases of spotted typhus. On the other hand, in modern times spotted typhus has followed every army which has moved out of the east into Central Europe. The severest epidemic which has recently visited Germany was brought with the French army in its retreat from Russia; the next pestilence of the sort which attacked a French army occurred, when on the soil of the Crimea they came into new contact with Sclavonic troops. The hunger pestilence of Upper Silesia in 1848, and of East Prussia in 1868, stood related in many respects to Sclavonic populations. Even in our province of Posen, there have been since 1828 a number of small epidemics of recurrent fever and spotted typhus. "Poland appears to have as dangerous a significance for us as Ireland for Great Britain; and much as I was formerly opposed to admitting contagion as the common means of the development of typhus-epidemics, I must confess that, like so many earlier observers, continued experience impels me more and more to go over to the camp of the contagionists."

The paper closes with the hope that the war may find scientific and well-instructed historians. "Every war is a heavy evil, and the real gain lies often enough in a very different direction from material acquisitions."—*New York Medical Record.*

